# Winner of the Ronald Melzack–*Canadian Journal of Pain* 2018 Paper of the Year Award/Récipiendaire du Prix Ronald Melzack pour L’Annee 2018 des Articles Parus dans *La Revue Canadienne de la Douleur*

**DOI:** 10.1080/24740527.2019.1622965

**Published:** 2019-06-12

**Authors:** Joel Katz

**Affiliations:** ^a^ Department of Psychology, York University; ^b^ Department of Anesthesia & Pain Management, Toronto General Hospital

This year’s prize for the Ronald Melzack–*Canadian Journal of Pain* 2018 Paper of the Year Award goes to Dr. Heather Hajistavropoulos and colleagues, Drs. Luke Schneider, Thomas Hadjistavropoulos, Nickolai Titov, and Blake Dear, for their article titled “Effectiveness, Acceptability and Feasibility of an Internet-Delivered Cognitive Behavioral Pain Management Program in a Routine Online Therapy Clinic in Canada.”^1^ Dr. Laura Stone, Canadian Pain Society (CPS) Awards Committee Chair, and I presented the award to co-author Dr. Thomas Hadjistavropoulos at the Canadian Pain Society’s Gala Dinner on April 4, 2018. The prize included a framed certificate from the CPS and Taylor & Francis, a ticket to the CPS Annual Meeting gala dinner, and a voucher waiving article processing fees for a manuscript to be submitted to the *Canadian Journal of Pain* in 2019. The winning paper was selected from among 23 original articles published in the *Journal* in 2018 and determined by the editor in conjunction with *Journal* Editorial Board members who considered originality, novelty, quality, and potential impact when submitting their votes.

The winning article combines the most efficacious, evidence-based treatment for chronic pain; namely, cognitive behavioral pain management,^2^ with a coach-supported, Internet delivery format that has the potential to reach the millions of Canadians who live with chronic pain on a daily basis but have little access to care. Recent randomized controlled trials have shown efficacy for the Internet-delivered Pain Course,^3,4^ but its effectiveness, acceptability, and feasibility had yet to be evaluated in a routine online clinical practice setting. What works under the antiseptic conditions of a clinical trial in which patients are carefully selected and treatment protocols are assiduously followed may not produce the same benefits under real-world conditions.^5^

The authors recruited 55 individuals with chronic pain, from the province of Saskatchewan, to participate in the Pain Course, which targets pain, disability, depression, and anxiety across an 8-week period using a series of five online lessons featuring slide shows that focus on cognitive behavioral therapy issues relevant to living and coping with chronic pain. The Pain Course was supplemented by support from a doctoral-level clinical psychology graduate student who served as coach and spent between 10 and 15 min each week speaking with clients by telephone. Participants were assessed at baseline before treatment began, posttreatment, and at follow-up 3 months after the end of treatment. Primary outcome measures included disability, depression, and anxiety. Secondary outcomes included pain severity, pain acceptance, pain self-efficacy, and kinesiophobia. The results showed significant improvement from baseline to posttreatment follow-up on all primary and secondary measures except pain self-efficacy and kinesiophobia. Importantly, 73% of clients who completed the Pain Course reported being satisfied or very satisfied with the program and 85% or more indicated that it was worth their time and/or they would recommend it to a friend. Over the 8-week trial period, the coach spent fewer than 2 h (approximately seven phone calls) and sent approximately 4.5 text messages per client. Taken together, this real-world evaluation indicates that the Pain Course is effective, acceptable, and feasible at least for use, as indicated, in the province of Saskatchewan. The minimal time spent by the support coach with each client is considerably less than that required even for in-person group psychotherapy, and although the authors did not include a formal cost analysis, it would appear that the Pain Course is less costly than traditional psychotherapy. The fact that 55% of clients lived in a rural setting or small cities hints at the potential benefits of expanding the online Pain Course intervention to these communities given the challenges and inequities associated with health care delivery in small cities and remote regions.^6^

The *Journal* Editorial Board and the CPS Awards Committee offer our hearty congratulations to Dr. Hadjistavropoulos and colleagues for their award-winning article. We look forward to next year’s competition when we expect to present one award for the basic/preclinical sciences and another for the applied/clinical sciences. We are grateful to the Canadian and international pain communities for supporting the *Journal* with manuscript submissions. Please continue to submit your original research to the *Canadian Journal of Pain* and become eligible for The Ronald Melzack–*Canadian Journal of Pain* 2019 Paper of the Year Award/Prix Ronald Melzack Pour L’Annee 2019 des Articles Parus dans *La Revue Canadienne de la Douleur*!
